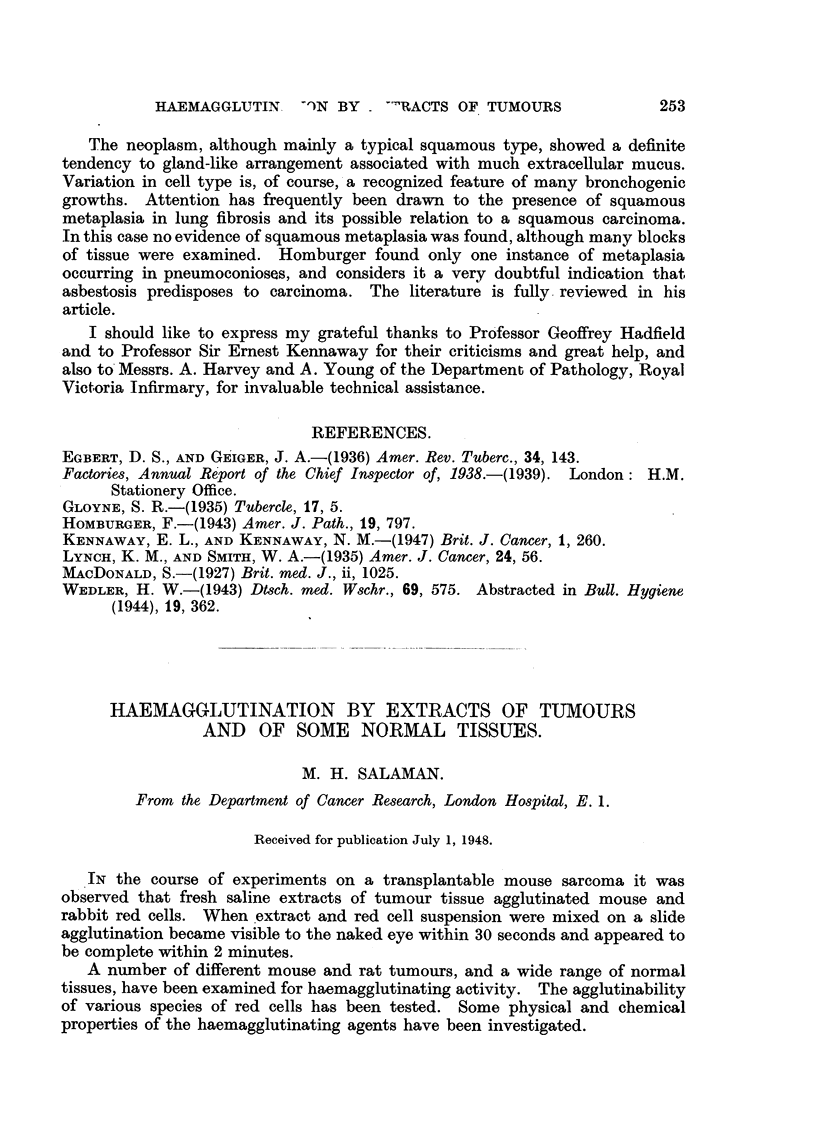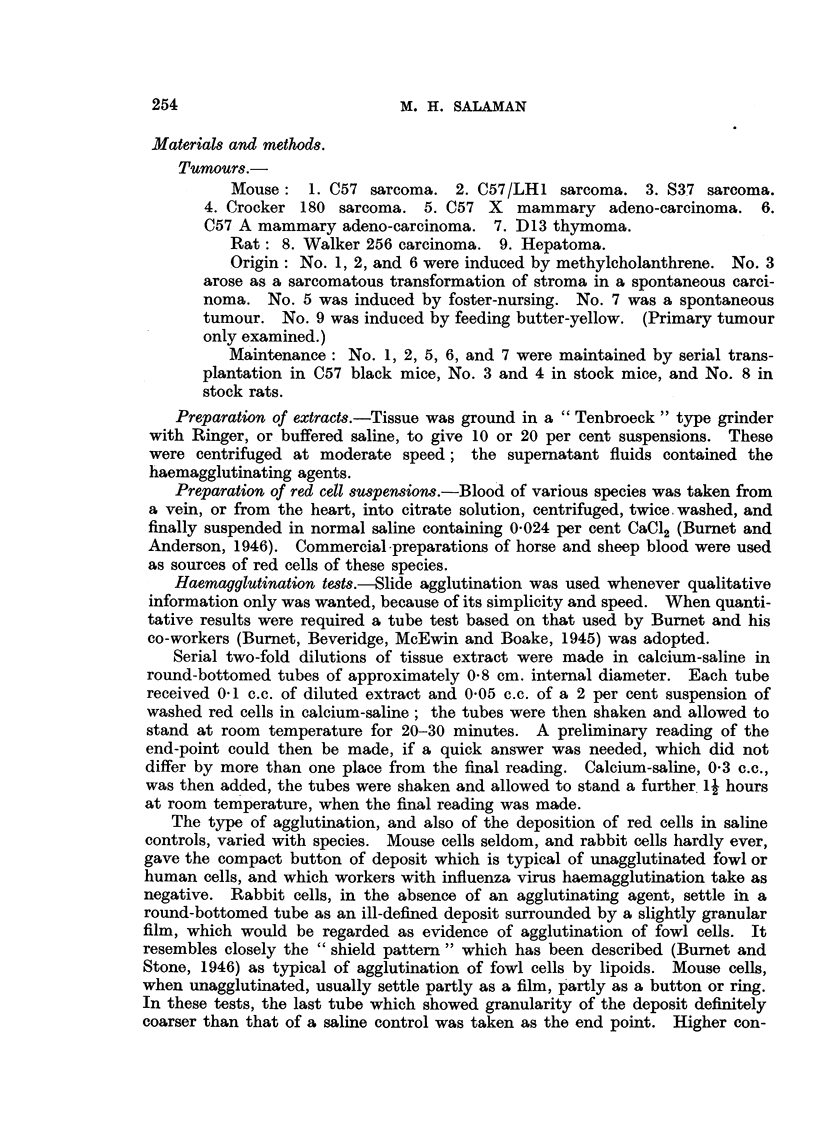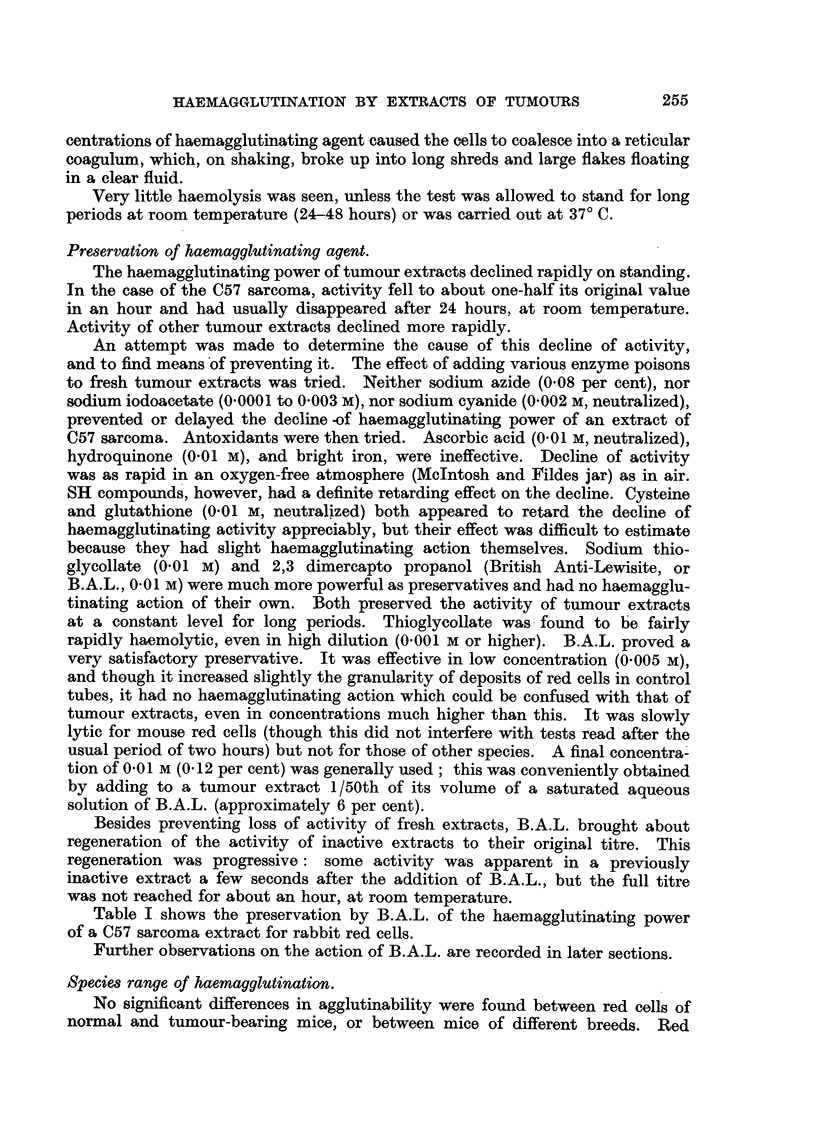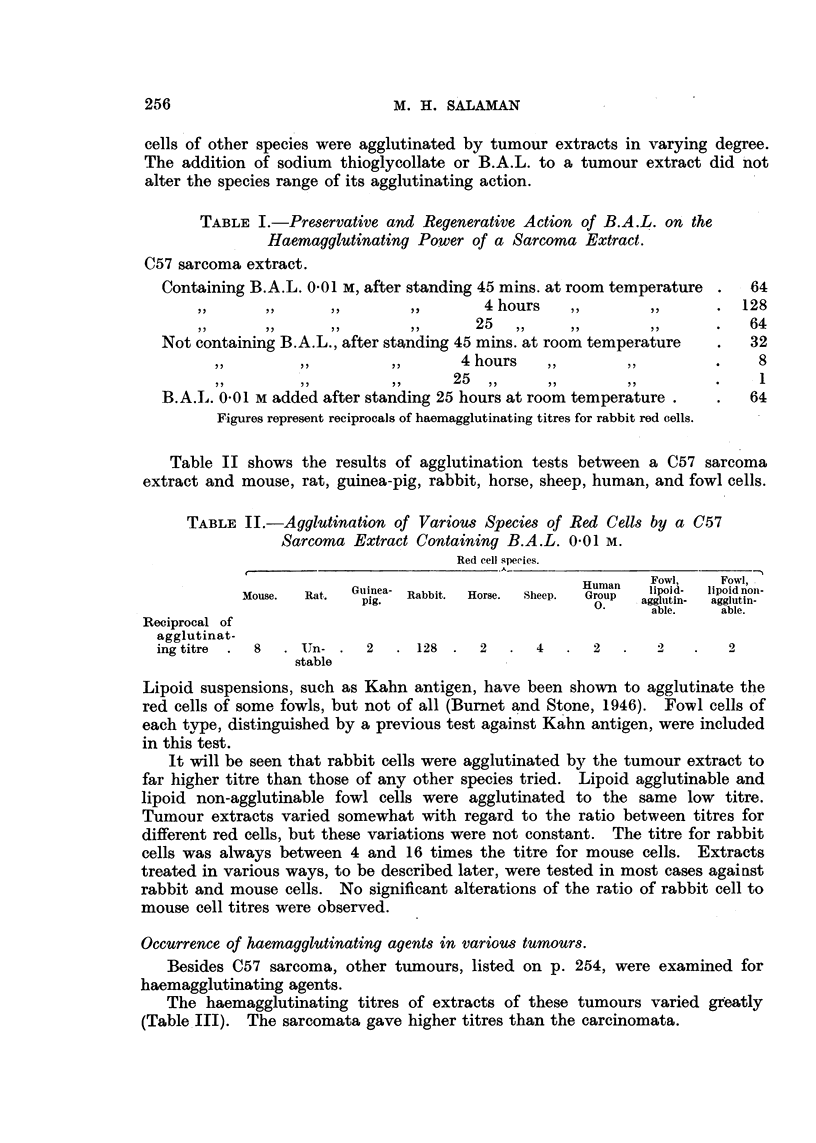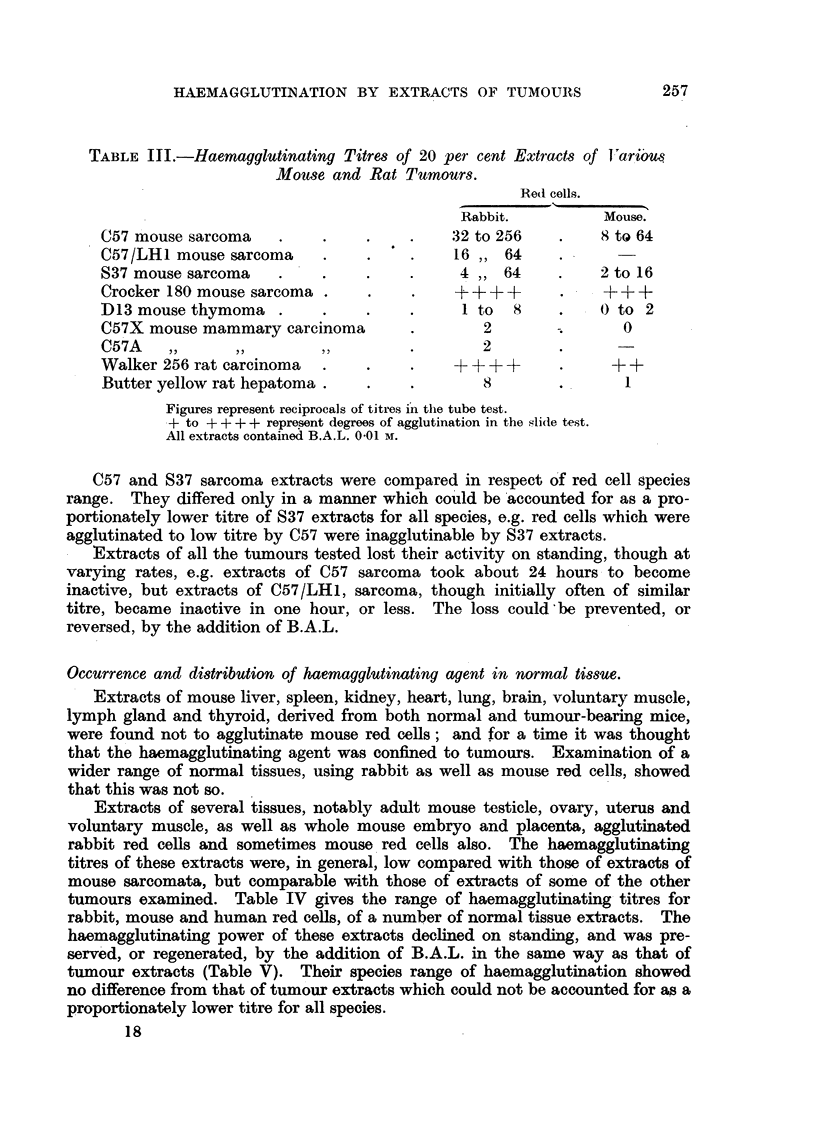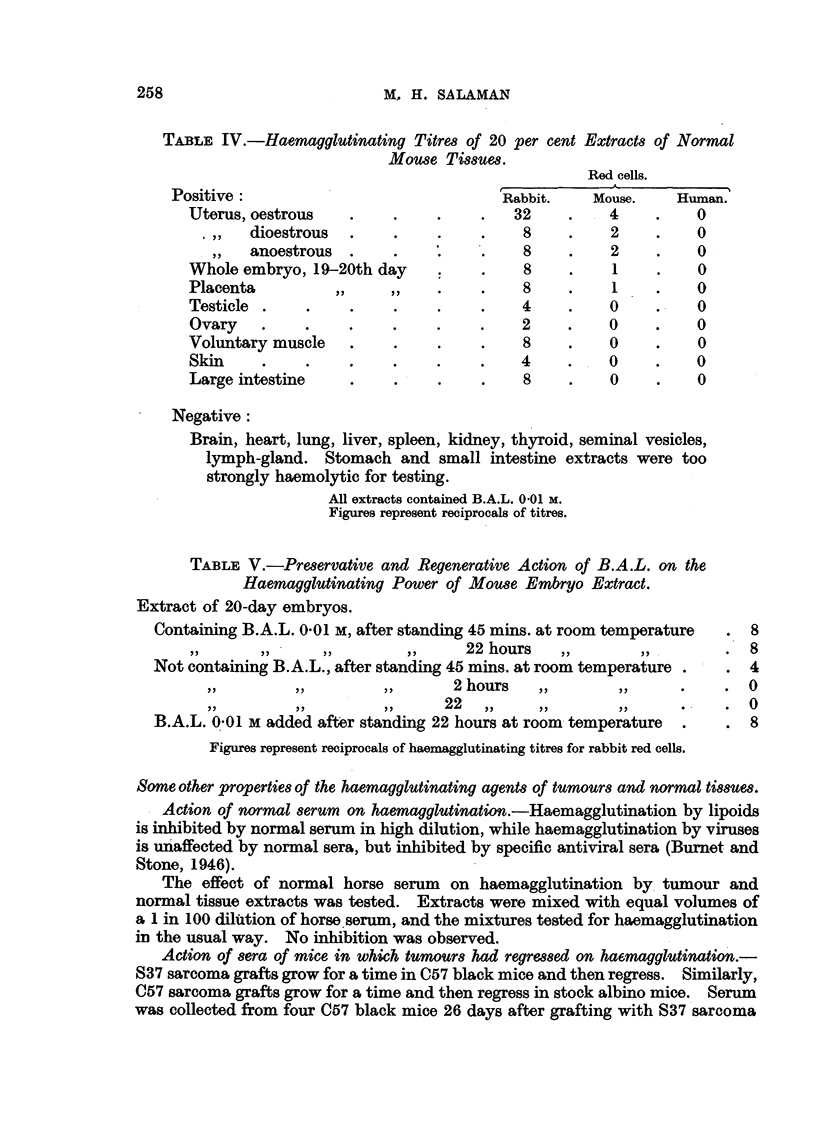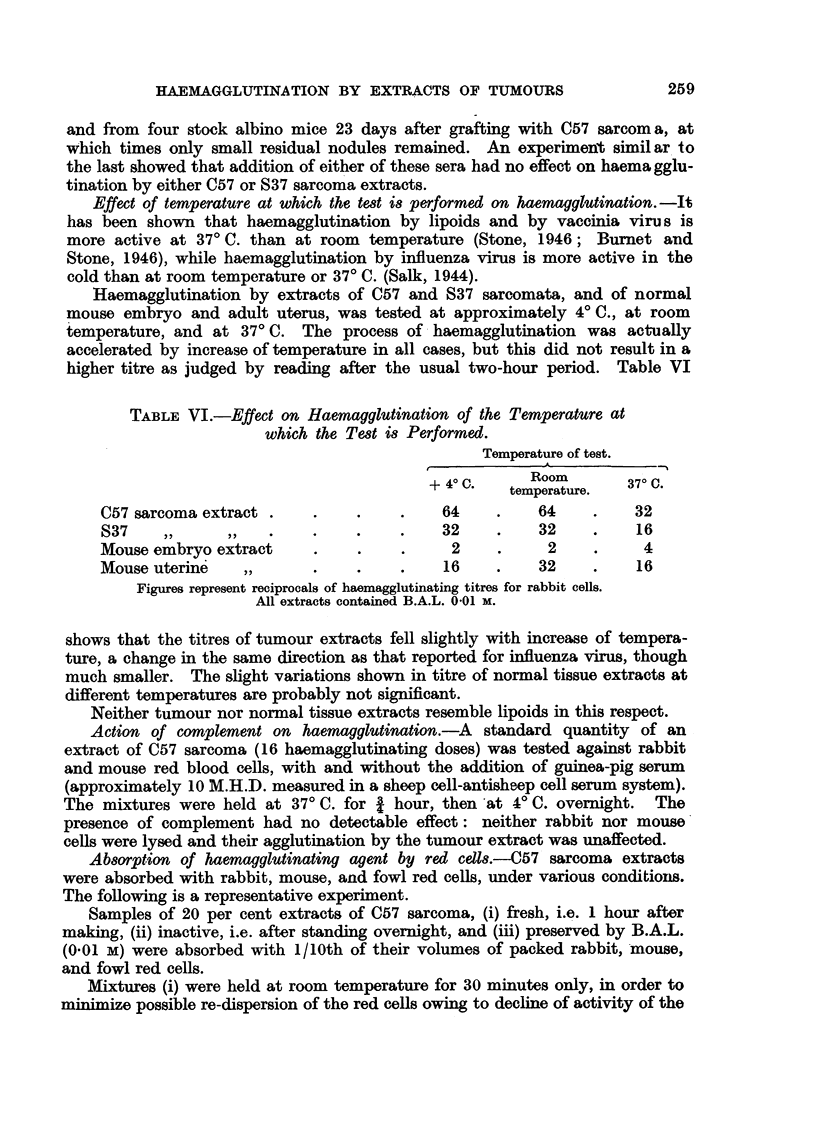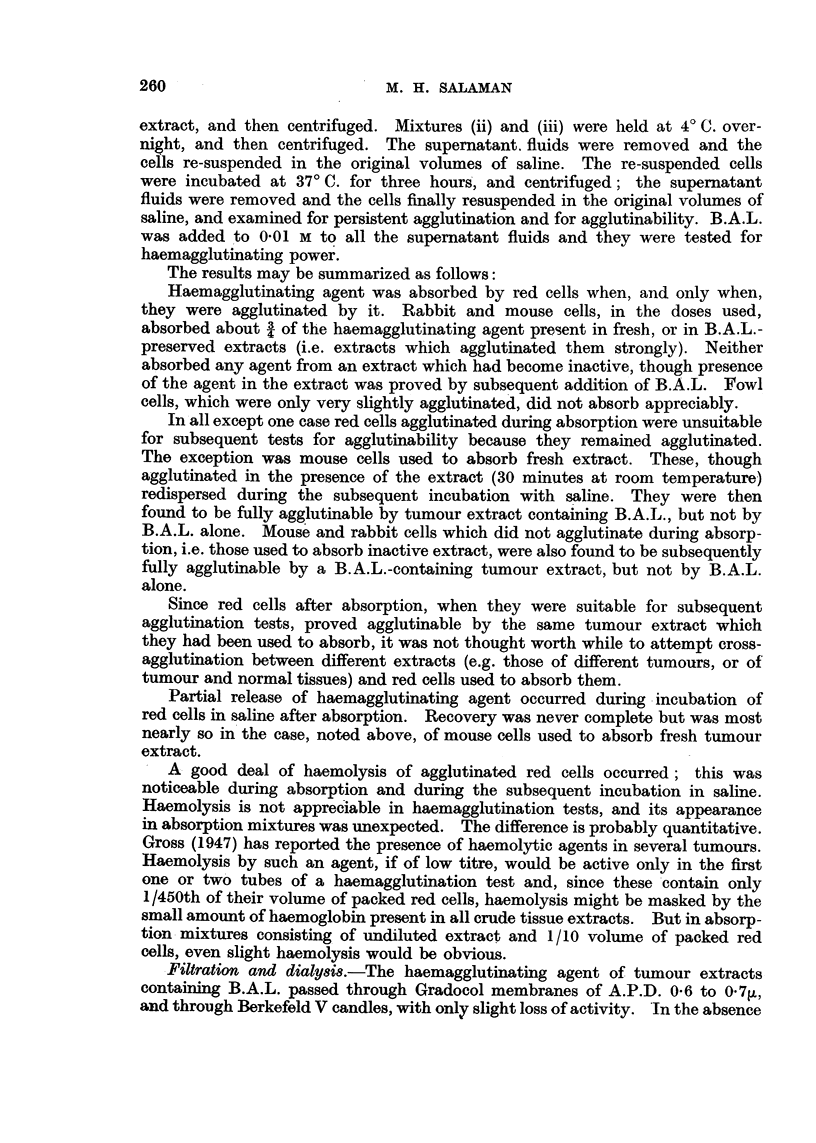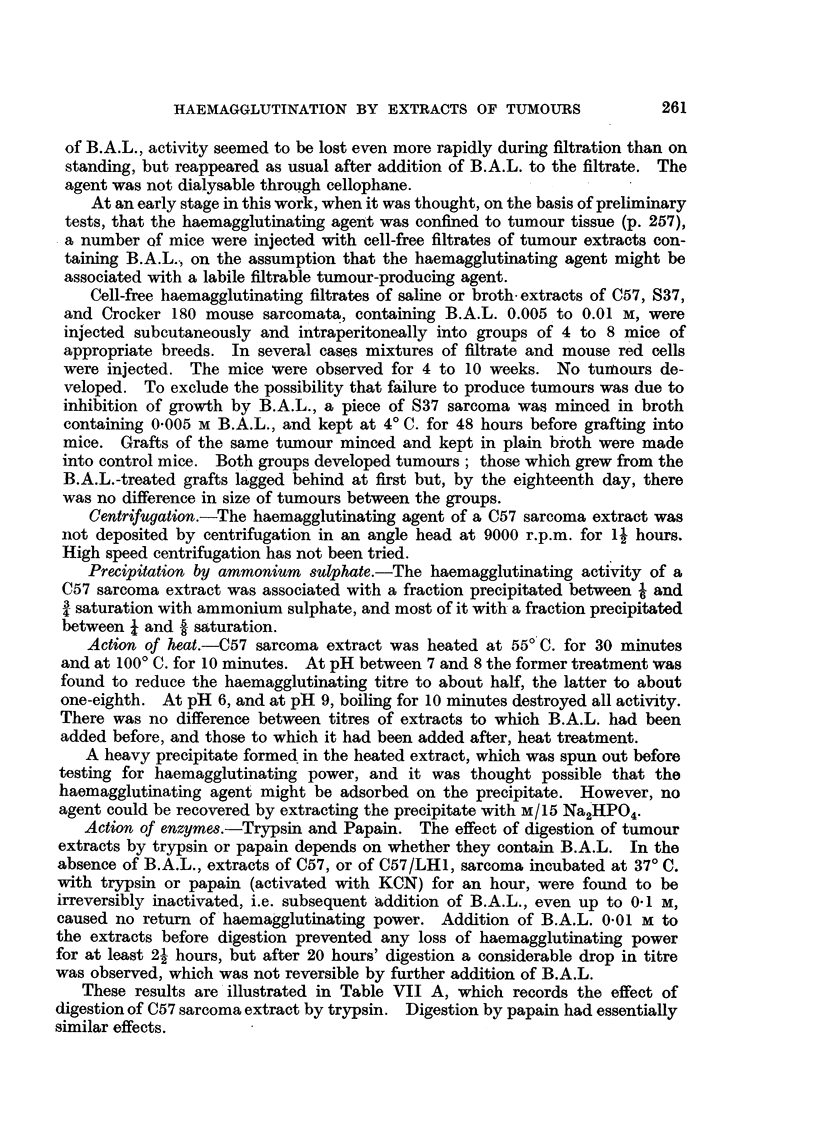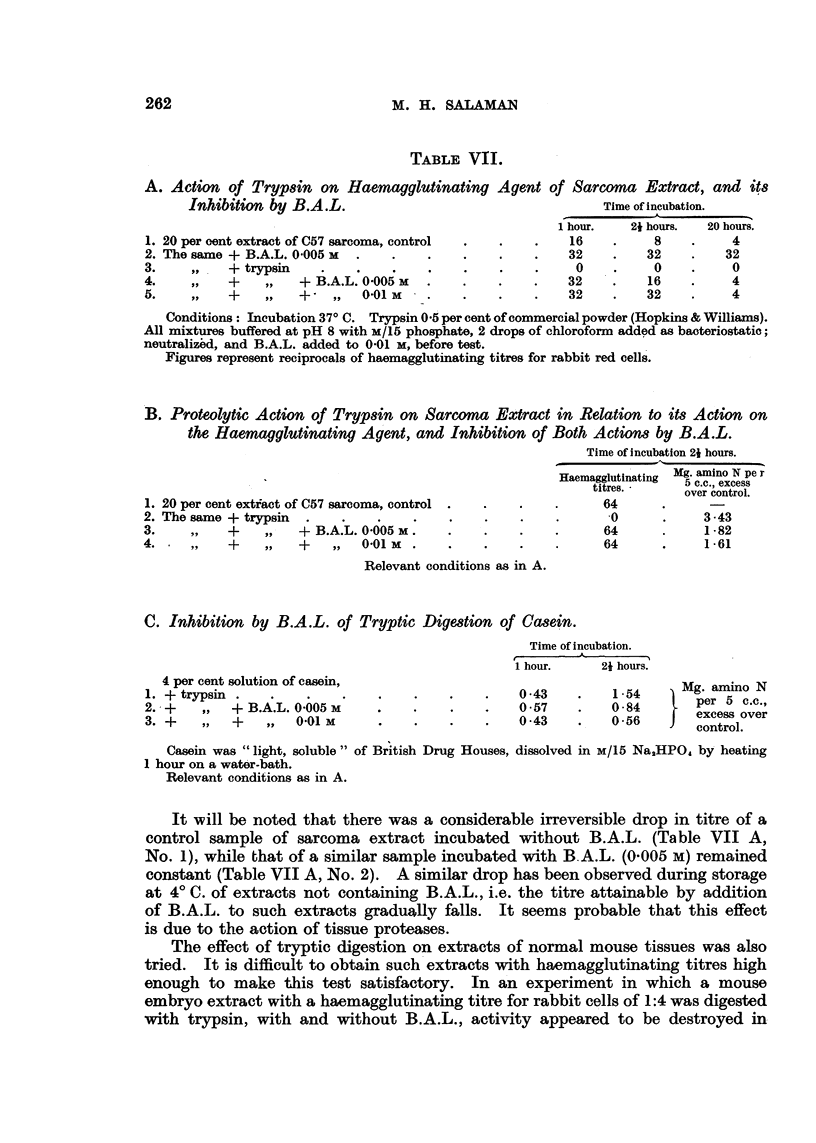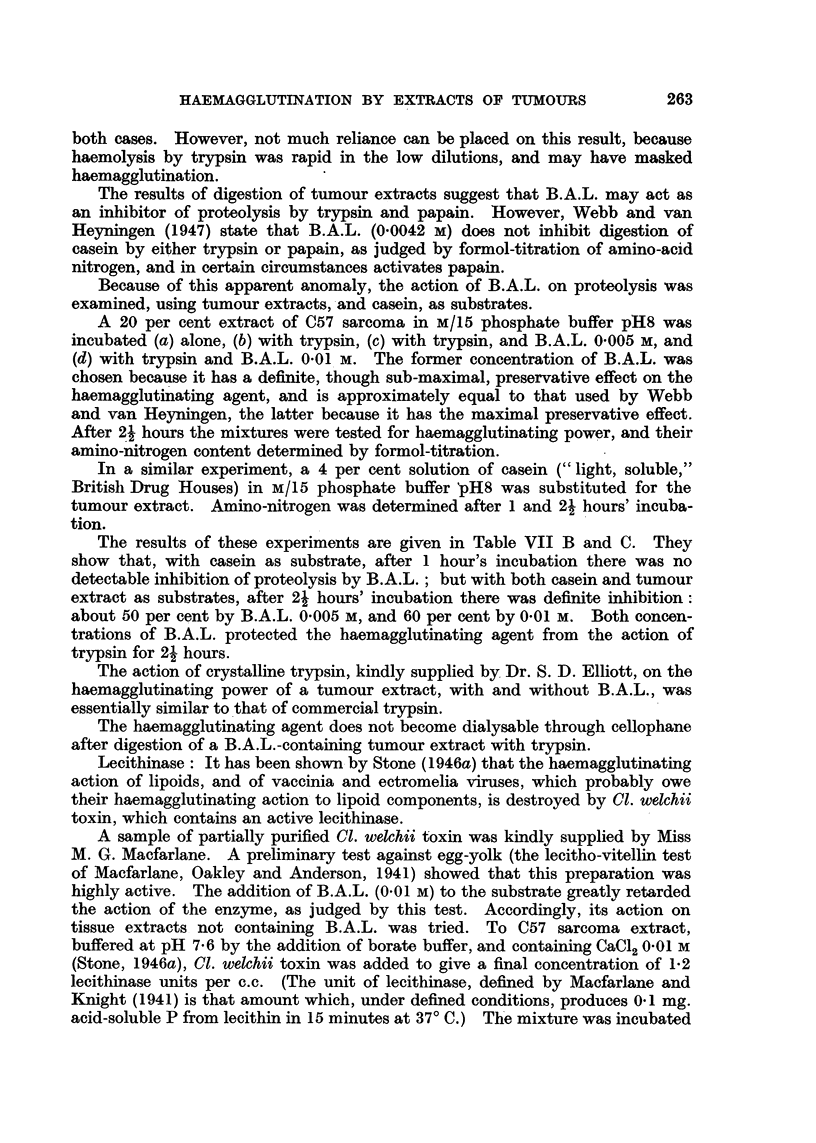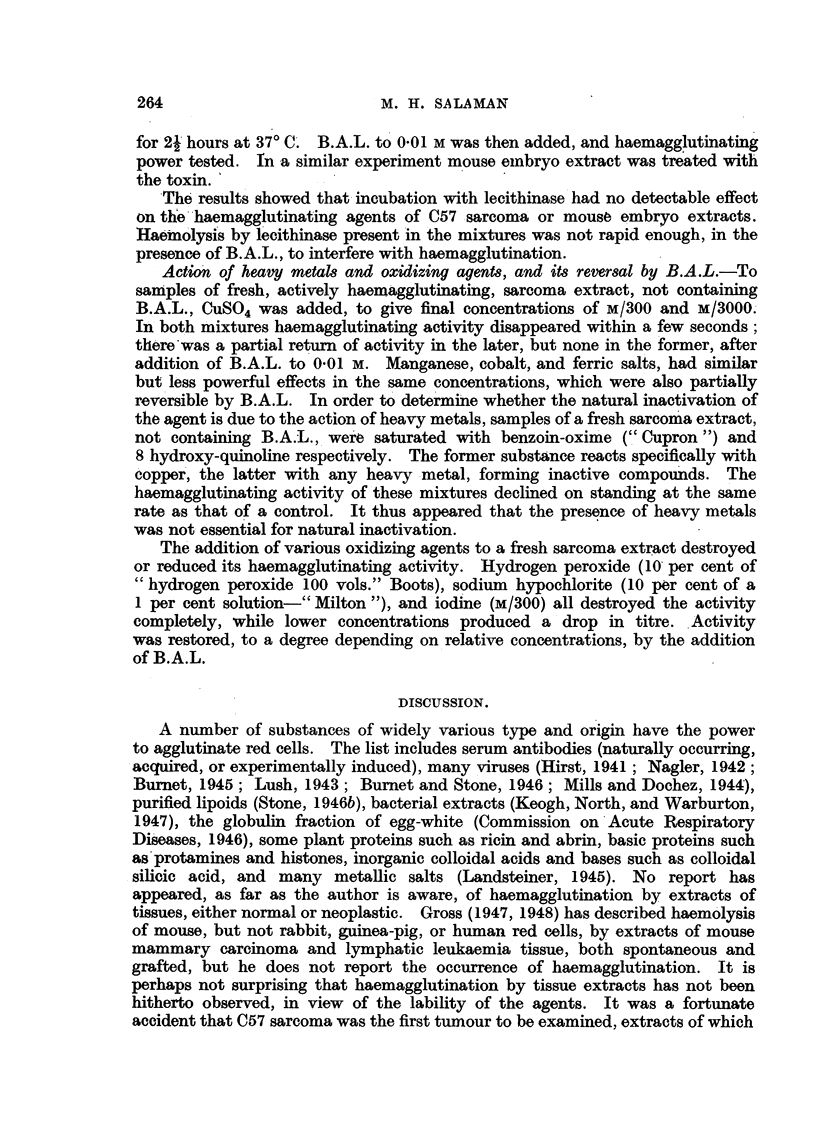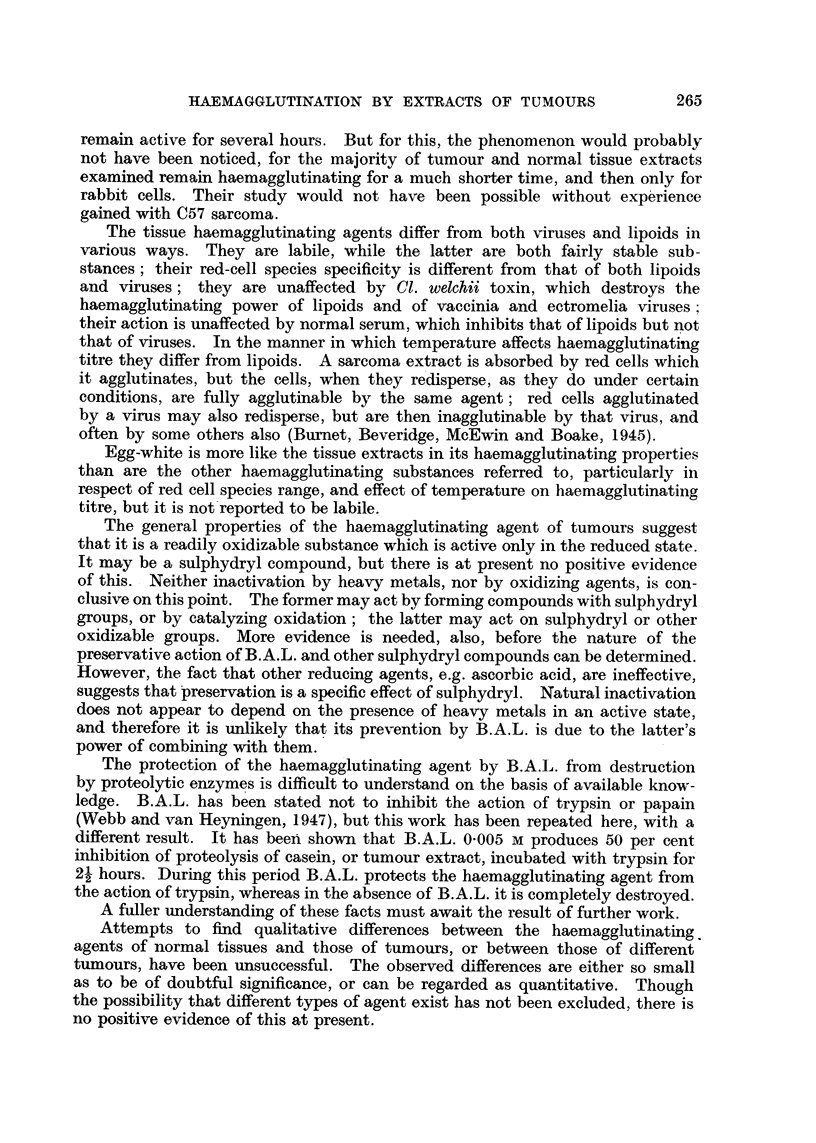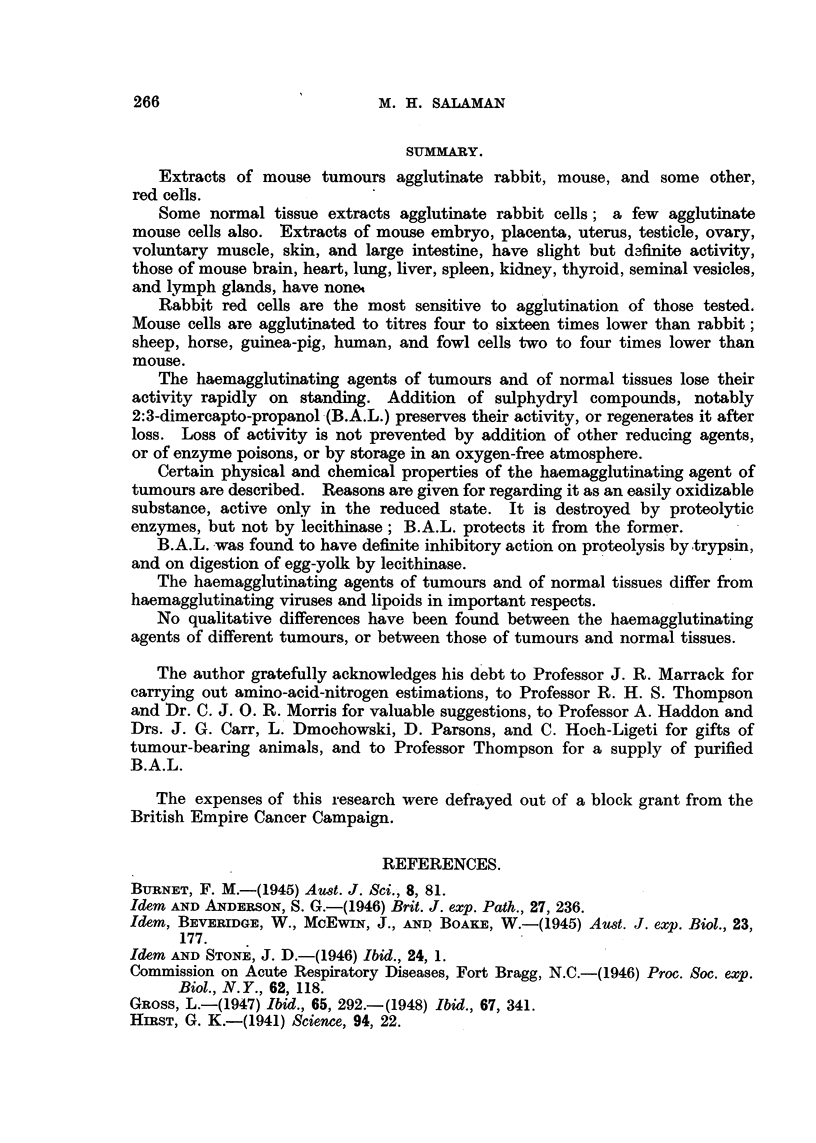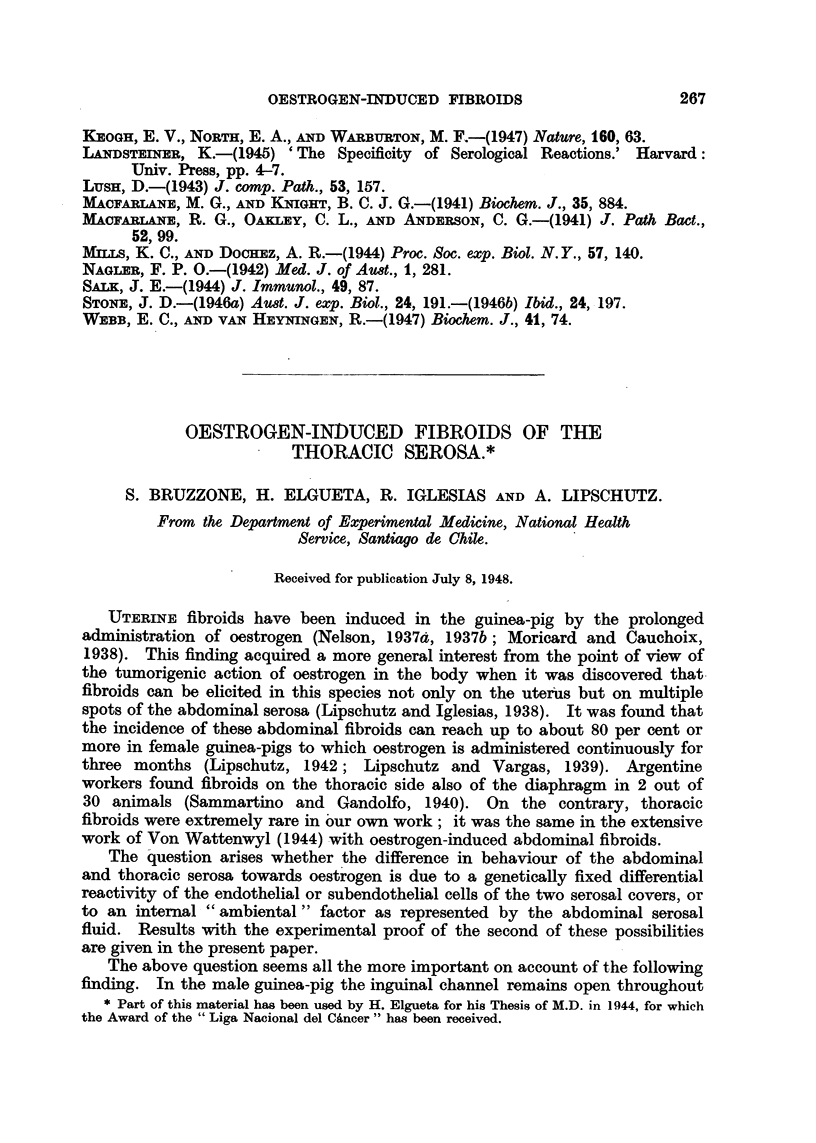# Haemagglutination by Extracts of Tumours and of Some Normal Tissues

**DOI:** 10.1038/bjc.1948.30

**Published:** 1948-09

**Authors:** M. H. Salaman


### HAEMAGGLUTINATION BY EXTRACTS OF TUMOURS AND OF SOME NORMAL TISSUES.

M. H. SALAMAN.

*From the Department of Cancer Research, London Hospital, E. 1.*

Received for publication July 1, 1948.

In the course of experiments on a transplantable mouse sarcoma it was observed that fresh saline extracts of tumour tissue agglutinated mouse and rabbit red cells. When extract and red cell suspension were mixed on a slide agglutination became visible to the naked eye within 30 seconds and appeared to be complete within 2 minutes.

A number of different mouse and rat tumours, and a wide range of normal tissues, have been examined for haemagglutinating activity. The agglutinability of various species of red cells has been tested. Some physical and chemical properties of the haemagglutinating agents have been investigated.

*Materials and methods.*

*Tumours.*—

Mouse: 1. C57 sarcoma. 2. C57/LH1 sarcoma. 3. S37 sarcoma. 4. Crocker 180 sarcoma. 5. C57 X mammary adeno-carcinoma. 6. C57 A mammary adeno-carcinoma. 7. D13 thymoma.

Rat: 8. Walker 256 carcinoma. 9. Hepatoma.

Origin: No. 1, 2, and 6 were induced by methylcholanthrene. No. 3 arose as a sarcomatous transformation of stroma in a spontaneous carcinoma. No. 5 was induced by foster-nursing. No. 7 was a spontaneous tumour. No. 9 was induced by feeding butter-yellow. (Primary tumour only examined.)

Maintenance: No. 1, 2, 5, 6, and 7 were maintained by serial transplantation in C57 black mice, No. 3 and 4 in stock mice, and No. 8 in stock rats.

*Preparation of extracts.*—Tissue was ground in a "Tenbroeck" type grinder with Ringer, or buffered saline, to give 10 or 20 per cent suspensions. These were centrifuged at moderate speed; the supernatant fluids contained the haemagglutinating agents.

*Preparation of red cell suspensions.*—Blood of various species was taken from a vein, or from the heart, into citrate solution, centrifuged, twice washed, and finally suspended in normal saline containing 0·024 per cent $CaCl_2$ (Burnet and Anderson, 1946). Commercial preparations of horse and sheep blood were used as sources of red cells of these species.

*Haemagglutination tests.*—Slide agglutination was used whenever qualitative information only was wanted, because of its simplicity and speed. When quantitative results were required a tube test based on that used by Burnet and his co-workers (Burnet, Beveridge, McEwin and Boake, 1945) was adopted.

Serial two-fold dilutions of tissue extract were made in calcium-saline in round-bottomed tubes of approximately 0·8 cm. internal diameter. Each tube received 0·1 c.c. of diluted extract and 0·05 c.c. of a 2 per cent suspension of washed red cells in calcium-saline; the tubes were then shaken and allowed to stand at room temperature for 20–30 minutes. A preliminary reading of the end-point could then be made, if a quick answer was needed, which did not differ by more than one place from the final reading. Calcium-saline, 0·3 c.c., was then added, the tubes were shaken and allowed to stand a further $1\frac{1}{2}$ hours at room temperature, when the final reading was made.

The type of agglutination, and also of the deposition of red cells in saline controls, varied with species. Mouse cells seldom, and rabbit cells hardly ever, gave the compact button of deposit which is typical of unagglutinated fowl or human cells, and which workers with influenza virus haemagglutination take as negative. Rabbit cells, in the absence of an agglutinating agent, settle in a round-bottomed tube as an ill-defined deposit surrounded by a slightly granular film, which would be regarded as evidence of agglutination of fowl cells. It resembles closely the "shield pattern" which has been described (Burnet and Stone, 1946) as typical of agglutination of fowl cells by lipoids. Mouse cells, when unagglutinated, usually settle partly as a film, partly as a button or ring. In these tests, the last tube which showed granularity of the deposit definitely coarser than that of a saline control was taken as the end point. Higher concentrations of haemagglutinating agent caused the cells to coalesce into a reticular coagulum, which, on shaking, broke up into long shreds and large flakes floating in a clear fluid.

Very little haemolysis was seen, unless the test was allowed to stand for long periods at room temperature (24–48 hours) or was carried out at 37° C.

*Preservation of haemagglutinating agent.*

The haemagglutinating power of tumour extracts declined rapidly on standing. In the case of the C57 sarcoma, activity fell to about one-half its original value in an hour and had usually disappeared after 24 hours, at room temperature. Activity of other tumour extracts declined more rapidly.

An attempt was made to determine the cause of this decline of activity, and to find means of preventing it. The effect of adding various enzyme poisons to fresh tumour extracts was tried. Neither sodium azide (0·08 per cent), nor sodium iodoacetate (0·0001 to 0·003 M), nor sodium cyanide (0·002 M, neutralized), prevented or delayed the decline of haemagglutinating power of an extract of C57 sarcoma. Antoxidants were then tried. Ascorbic acid (0·01 M, neutralized), hydroquinone (0·01 M), and bright iron, were ineffective. Decline of activity was as rapid in an oxygen-free atmosphere (McIntosh and Fildes jar) as in air. SH compounds, however, had a definite retarding effect on the decline. Cysteine and glutathione (0·01 M, neutralized) both appeared to retard the decline of haemagglutinating activity appreciably, but their effect was difficult to estimate because they had slight haemagglutinating action themselves. Sodium thioglycollate (0·01 M) and 2,3 dimercapto propanol (British Anti-Lewisite, or B.A.L., 0·01 M) were much more powerful as preservatives and had no haemagglutinating action of their own. Both preserved the activity of tumour extracts at a constant level for long periods. Thioglycollate was found to be fairly rapidly haemolytic, even in high dilution (0·001 M or higher). B.A.L. proved a very satisfactory preservative. It was effective in low concentration (0·005 M), and though it increased slightly the granularity of deposits of red cells in control tubes, it had no haemagglutinating action which could be confused with that of tumour extracts, even in concentrations much higher than this. It was slowly lytic for mouse red cells (though this did not interfere with tests read after the usual period of two hours) but not for those of other species. A final concentration of 0·01 M (0·12 per cent) was generally used ; this was conveniently obtained by adding to a tumour extract 1/50th of its volume of a saturated aqueous solution of B.A.L. (approximately 6 per cent).

Besides preventing loss of activity of fresh extracts, B.A.L. brought about regeneration of the activity of inactive extracts to their original titre. This regeneration was progressive : some activity was apparent in a previously inactive extract a few seconds after the addition of B.A.L., but the full titre was not reached for about an hour, at room temperature.

Table I shows the preservation by B.A.L. of the haemagglutinating power of a C57 sarcoma extract for rabbit red cells.

Further observations on the action of B.A.L. are recorded in later sections.

*Species range of haemagglutination.*

No significant differences in agglutinability were found between red cells of normal and tumour-bearing mice, or between mice of different breeds. Red cells of other species were agglutinated by tumour extracts in varying degree. The addition of sodium thioglycollate or B.A.L. to a tumour extract did not alter the species range of its agglutinating action.

TABLE I.—*Preservative and Regenerative Action of B.A.L. on the Haemagglutinating Power of a Sarcoma Extract.*

C57 sarcoma extract.

| | |
|---|---|
| Containing B.A.L. 0·01 M, after standing 45 mins. at room temperature | 64 |
| ,, ,, ,, ,, 4 hours ,, ,, | 128 |
| ,, ,, ,, ,, 25 ,, ,, ,, | 64 |
| Not containing B.A.L., after standing 45 mins. at room temperature | 32 |
| ,, ,, ,, 4 hours ,, ,, | 8 |
| ,, ,, ,, 25 ,, ,, ,, | 1 |
| B.A.L. 0·01 M added after standing 25 hours at room temperature | 64 |

Figures represent reciprocals of haemagglutinating titres for rabbit red cells.

Table II shows the results of agglutination tests between a C57 sarcoma extract and mouse, rat, guinea-pig, rabbit, horse, sheep, human, and fowl cells.

TABLE II.—*Agglutination of Various Species of Red Cells by a C57 Sarcoma Extract Containing B.A.L.* 0·01 M.

| | Red cell species. | | | | | | | | |
|---|---|---|---|---|---|---|---|---|---|
| | Mouse. | Rat. | Guinea-pig. | Rabbit. | Horse. | Sheep. | Human Group O. | Fowl, lipoid-agglutinable. | Fowl, lipoid non-agglutinable. |
| Reciprocal of agglutinating titre | 8 | Unstable | 2 | 128 | 2 | 4 | 2 | 2 | 2 |

Lipoid suspensions, such as Kahn antigen, have been shown to agglutinate the red cells of some fowls, but not of all (Burnet and Stone, 1946). Fowl cells of each type, distinguished by a previous test against Kahn antigen, were included in this test.

It will be seen that rabbit cells were agglutinated by the tumour extract to far higher titre than those of any other species tried. Lipoid agglutinable and lipoid non-agglutinable fowl cells were agglutinated to the same low titre. Tumour extracts varied somewhat with regard to the ratio between titres for different red cells, but these variations were not constant. The titre for rabbit cells was always between 4 and 16 times the titre for mouse cells. Extracts treated in various ways, to be described later, were tested in most cases against rabbit and mouse cells. No significant alterations of the ratio of rabbit cell to mouse cell titres were observed.

*Occurrence of haemagglutinating agents in various tumours.*

Besides C57 sarcoma, other tumours, listed on p. 254, were examined for haemagglutinating agents.

The haemagglutinating titres of extracts of these tumours varied greatly (Table III). The sarcomata gave higher titres than the carcinomata.

TABLE III.—*Haemagglutinating Titres of 20 per cent Extracts of Various Mouse and Rat Tumours.*

| | Red cells. | |
|---|---|---|
| | Rabbit. | Mouse. |
| C57 mouse sarcoma | 32 to 256 | 8 to 64 |
| C57/LH1 mouse sarcoma | 16 ,, 64 | — |
| S37 mouse sarcoma | 4 ,, 64 | 2 to 16 |
| Crocker 180 mouse sarcoma | ++++ | +++ |
| D13 mouse thymoma | 1 to 8 | 0 to 2 |
| C57X mouse mammary carcinoma | 2 | 0 |
| C57A ,, ,, ,, | 2 | — |
| Walker 256 rat carcinoma | ++++ | ++ |
| Butter yellow rat hepatoma | 8 | 1 |

Figures represent reciprocals of titres in the tube test.
+ to ++++ represent degrees of agglutination in the slide test.
All extracts contained B.A.L. 0·01 M.

C57 and S37 sarcoma extracts were compared in respect of red cell species range. They differed only in a manner which could be accounted for as a proportionately lower titre of S37 extracts for all species, e.g. red cells which were agglutinated to low titre by C57 were inagglutinable by S37 extracts.

Extracts of all the tumours tested lost their activity on standing, though at varying rates, e.g. extracts of C57 sarcoma took about 24 hours to become inactive, but extracts of C57/LH1, sarcoma, though initially often of similar titre, became inactive in one hour, or less. The loss could be prevented, or reversed, by the addition of B.A.L.

*Occurrence and distribution of haemagglutinating agent in normal tissue.*

Extracts of mouse liver, spleen, kidney, heart, lung, brain, voluntary muscle, lymph gland and thyroid, derived from both normal and tumour-bearing mice, were found not to agglutinate mouse red cells; and for a time it was thought that the haemagglutinating agent was confined to tumours. Examination of a wider range of normal tissues, using rabbit as well as mouse red cells, showed that this was not so.

Extracts of several tissues, notably adult mouse testicle, ovary, uterus and voluntary muscle, as well as whole mouse embryo and placenta, agglutinated rabbit red cells and sometimes mouse red cells also. The haemagglutinating titres of these extracts were, in general, low compared with those of extracts of mouse sarcomata, but comparable with those of extracts of some of the other tumours examined. Table IV gives the range of haemagglutinating titres for rabbit, mouse and human red cells, of a number of normal tissue extracts. The haemagglutinating power of these extracts declined on standing, and was preserved, or regenerated, by the addition of B.A.L. in the same way as that of tumour extracts (Table V). Their species range of haemagglutination showed no difference from that of tumour extracts which could not be accounted for as a proportionately lower titre for all species.

TABLE IV.—*Haemagglutinating Titres of 20 per cent Extracts of Normal Mouse Tissues.*

| | Red cells. | | |
|---|---|---|---|
| | Rabbit. | Mouse. | Human. |
| Positive : | | | |
| Uterus, oestrous | 32 | 4 | 0 |
| ,, dioestrous | 8 | 2 | 0 |
| ,, anoestrous | 8 | 2 | 0 |
| Whole embryo, 19–20th day | 8 | 1 | 0 |
| Placenta ,, ,, | 8 | 1 | 0 |
| Testicle | 4 | 0 | 0 |
| Ovary | 2 | 0 | 0 |
| Voluntary muscle | 8 | 0 | 0 |
| Skin | 4 | 0 | 0 |
| Large intestine | 8 | 0 | 0 |

Negative :

Brain, heart, lung, liver, spleen, kidney, thyroid, seminal vesicles, lymph-gland. Stomach and small intestine extracts were too strongly haemolytic for testing.

All extracts contained B.A.L. 0·01 M.
Figures represent reciprocals of titres.

TABLE V.—*Preservative and Regenerative Action of B.A.L. on the Haemagglutinating Power of Mouse Embryo Extract.*

Extract of 20-day embryos.

| | |
|---|---|
| Containing B.A.L. 0·01 M, after standing 45 mins. at room temperature | 8 |
| ,, ,, ,, ,, 22 hours ,, ,, | 8 |
| Not containing B.A.L., after standing 45 mins. at room temperature | 4 |
| ,, ,, ,, 2 hours ,, ,, | 0 |
| ,, ,, ,, 22 ,, ,, ,, | 0 |
| B.A.L. 0·01 M added after standing 22 hours at room temperature | 8 |

Figures represent reciprocals of haemagglutinating titres for rabbit red cells.

*Some other properties of the haemagglutinating agents of tumours and normal tissues.*

*Action of normal serum on haemagglutination.*—Haemagglutination by lipoids is inhibited by normal serum in high dilution, while haemagglutination by viruses is unaffected by normal sera, but inhibited by specific antiviral sera (Burnet and Stone, 1946).

The effect of normal horse serum on haemagglutination by tumour and normal tissue extracts was tested. Extracts were mixed with equal volumes of a 1 in 100 dilution of horse serum, and the mixtures tested for haemagglutination in the usual way. No inhibition was observed.

*Action of sera of mice in which tumours had regressed on haemagglutination.*—S37 sarcoma grafts grow for a time in C57 black mice and then regress. Similarly, C57 sarcoma grafts grow for a time and then regress in stock albino mice. Serum was collected from four C57 black mice 26 days after grafting with S37 sarcoma and from four stock albino mice 23 days after grafting with C57 sarcoma, at which times only small residual nodules remained. An experiment similar to the last showed that addition of either of these sera had no effect on haemagglutination by either C57 or S37 sarcoma extracts.

*Effect of temperature at which the test is performed on haemagglutination.*—It has been shown that haemagglutination by lipoids and by vaccinia virus is more active at 37° C. than at room temperature (Stone, 1946; Burnet and Stone, 1946), while haemagglutination by influenza virus is more active in the cold than at room temperature or 37° C. (Salk, 1944).

Haemagglutination by extracts of C57 and S37 sarcomata, and of normal mouse embryo and adult uterus, was tested at approximately 4° C., at room temperature, and at 37° C. The process of haemagglutination was actually accelerated by increase of temperature in all cases, but this did not result in a higher titre as judged by reading after the usual two-hour period. Table VI

TABLE VI.—*Effect on Haemagglutination of the Temperature at which the Test is Performed.*

| | Temperature of test. | | |
|---|---|---|---|
| | + 4° C. | Room temperature. | 37° C. |
| C57 sarcoma extract | 64 | 64 | 32 |
| S37 " " | 32 | 32 | 16 |
| Mouse embryo extract | 2 | 2 | 4 |
| Mouse uterine " | 16 | 32 | 16 |

Figures represent reciprocals of haemagglutinating titres for rabbit cells. All extracts contained B.A.L. 0·01 M.

shows that the titres of tumour extracts fell slightly with increase of temperature, a change in the same direction as that reported for influenza virus, though much smaller. The slight variations shown in titre of normal tissue extracts at different temperatures are probably not significant.

Neither tumour nor normal tissue extracts resemble lipoids in this respect.

*Action of complement on haemagglutination.*—A standard quantity of an extract of C57 sarcoma (16 haemagglutinating doses) was tested against rabbit and mouse red blood cells, with and without the addition of guinea-pig serum (approximately 10 M.H.D. measured in a sheep cell-antisheep cell serum system). The mixtures were held at 37° C. for $\frac{3}{4}$ hour, then at 4° C. overnight. The presence of complement had no detectable effect: neither rabbit nor mouse cells were lysed and their agglutination by the tumour extract was unaffected.

*Absorption of haemagglutinating agent by red cells.*—C57 sarcoma extracts were absorbed with rabbit, mouse, and fowl red cells, under various conditions. The following is a representative experiment.

Samples of 20 per cent extracts of C57 sarcoma, (i) fresh, i.e. 1 hour after making, (ii) inactive, i.e. after standing overnight, and (iii) preserved by B.A.L. (0·01 M) were absorbed with 1/10th of their volumes of packed rabbit, mouse, and fowl red cells.

Mixtures (i) were held at room temperature for 30 minutes only, in order to minimize possible re-dispersion of the red cells owing to decline of activity of the extract, and then centrifuged. Mixtures (ii) and (iii) were held at 4° C. overnight, and then centrifuged. The supernatant fluids were removed and the cells re-suspended in the original volumes of saline. The re-suspended cells were incubated at 37° C. for three hours, and centrifuged; the supernatant fluids were removed and the cells finally resuspended in the original volumes of saline, and examined for persistent agglutination and for agglutinability. B.A.L. was added to 0·01 M to all the supernatant fluids and they were tested for haemagglutinating power.

The results may be summarized as follows:

Haemagglutinating agent was absorbed by red cells when, and only when, they were agglutinated by it. Rabbit and mouse cells, in the doses used, absorbed about $\frac{3}{4}$ of the haemagglutinating agent present in fresh, or in B.A.L.-preserved extracts (i.e. extracts which agglutinated them strongly). Neither absorbed any agent from an extract which had become inactive, though presence of the agent in the extract was proved by subsequent addition of B.A.L. Fowl cells, which were only very slightly agglutinated, did not absorb appreciably.

In all except one case red cells agglutinated during absorption were unsuitable for subsequent tests for agglutinability because they remained agglutinated. The exception was mouse cells used to absorb fresh extract. These, though agglutinated in the presence of the extract (30 minutes at room temperature) redispersed during the subsequent incubation with saline. They were then found to be fully agglutinable by tumour extract containing B.A.L., but not by B.A.L. alone. Mouse and rabbit cells which did not agglutinate during absorption, i.e. those used to absorb inactive extract, were also found to be subsequently fully agglutinable by a B.A.L.-containing tumour extract, but not by B.A.L. alone.

Since red cells after absorption, when they were suitable for subsequent agglutination tests, proved agglutinable by the same tumour extract which they had been used to absorb, it was not thought worth while to attempt cross-agglutination between different extracts (e.g. those of different tumours, or of tumour and normal tissues) and red cells used to absorb them.

Partial release of haemagglutinating agent occurred during incubation of red cells in saline after absorption. Recovery was never complete but was most nearly so in the case, noted above, of mouse cells used to absorb fresh tumour extract.

A good deal of haemolysis of agglutinated red cells occurred; this was noticeable during absorption and during the subsequent incubation in saline. Haemolysis is not appreciable in haemagglutination tests, and its appearance in absorption mixtures was unexpected. The difference is probably quantitative. Gross (1947) has reported the presence of haemolytic agents in several tumours. Haemolysis by such an agent, if of low titre, would be active only in the first one or two tubes of a haemagglutination test and, since these contain only 1/450th of their volume of packed red cells, haemolysis might be masked by the small amount of haemoglobin present in all crude tissue extracts. But in absorption mixtures consisting of undiluted extract and 1/10 volume of packed red cells, even slight haemolysis would be obvious.

*Filtration and dialysis.*—The haemagglutinating agent of tumour extracts containing B.A.L. passed through Gradocol membranes of A.P.D. 0·6 to 0·7$\mu$, and through Berkefeld V candles, with only slight loss of activity. In the absence of B.A.L., activity seemed to be lost even more rapidly during filtration than on standing, but reappeared as usual after addition of B.A.L. to the filtrate. The agent was not dialysable through cellophane.

At an early stage in this work, when it was thought, on the basis of preliminary tests, that the haemagglutinating agent was confined to tumour tissue (p. 257), a number of mice were injected with cell-free filtrates of tumour extracts containing B.A.L., on the assumption that the haemagglutinating agent might be associated with a labile filtrable tumour-producing agent.

Cell-free haemagglutinating filtrates of saline or broth-extracts of C57, S37, and Crocker 180 mouse sarcomata, containing B.A.L. 0.005 to 0.01 M, were injected subcutaneously and intraperitoneally into groups of 4 to 8 mice of appropriate breeds. In several cases mixtures of filtrate and mouse red cells were injected. The mice were observed for 4 to 10 weeks. No tumours developed. To exclude the possibility that failure to produce tumours was due to inhibition of growth by B.A.L., a piece of S37 sarcoma was minced in broth containing 0·005 M B.A.L., and kept at 4° C. for 48 hours before grafting into mice. Grafts of the same tumour minced and kept in plain broth were made into control mice. Both groups developed tumours ; those which grew from the B.A.L.-treated grafts lagged behind at first but, by the eighteenth day, there was no difference in size of tumours between the groups.

*Centrifugation.*—The haemagglutinating agent of a C57 sarcoma extract was not deposited by centrifugation in an angle head at 9000 r.p.m. for $1\frac{1}{2}$ hours. High speed centrifugation has not been tried.

*Precipitation by ammonium sulphate.*—The haemagglutinating activity of a C57 sarcoma extract was associated with a fraction precipitated between $\frac{1}{6}$ and $\frac{3}{4}$ saturation with ammonium sulphate, and most of it with a fraction precipitated between $\frac{1}{4}$ and $\frac{5}{8}$ saturation.

*Action of heat.*—C57 sarcoma extract was heated at 55° C. for 30 minutes and at 100° C. for 10 minutes. At pH between 7 and 8 the former treatment was found to reduce the haemagglutinating titre to about half, the latter to about one-eighth. At pH 6, and at pH 9, boiling for 10 minutes destroyed all activity. There was no difference between titres of extracts to which B.A.L. had been added before, and those to which it had been added after, heat treatment.

A heavy precipitate formed in the heated extract, which was spun out before testing for haemagglutinating power, and it was thought possible that the haemagglutinating agent might be adsorbed on the precipitate. However, no agent could be recovered by extracting the precipitate with M/15 $Na_2HPO_4$.

*Action of enzymes.*—Trypsin and Papain. The effect of digestion of tumour extracts by trypsin or papain depends on whether they contain B.A.L. In the absence of B.A.L., extracts of C57, or of C57/LH1, sarcoma incubated at 37° C. with trypsin or papain (activated with KCN) for an hour, were found to be irreversibly inactivated, i.e. subsequent addition of B.A.L., even up to 0·1 M, caused no return of haemagglutinating power. Addition of B.A.L. 0·01 M to the extracts before digestion prevented any loss of haemagglutinating power for at least $2\frac{1}{2}$ hours, but after 20 hours' digestion a considerable drop in titre was observed, which was not reversible by further addition of B.A.L.

These results are illustrated in Table VII A, which records the effect of digestion of C57 sarcoma extract by trypsin. Digestion by papain had essentially similar effects.

TABLE VII.

A. *Action of Trypsin on Haemagglutinating Agent of Sarcoma Extract, and its Inhibition by B.A.L.*

| | Time of incubation. 1 hour. | 2½ hours. | 20 hours. |
|---|---|---|---|
| 1. 20 per cent extract of C57 sarcoma, control | 16 | 8 | 4 |
| 2. The same + B.A.L. 0·005 M | 32 | 32 | 32 |
| 3. ,, + trypsin | 0 | 0 | 0 |
| 4. ,, + ,, + B.A.L. 0·005 M | 32 | 16 | 4 |
| 5. ,, + ,, + ,, 0·01 M | 32 | 32 | 4 |

Conditions: Incubation 37° C. Trypsin 0·5 per cent of commercial powder (Hopkins & Williams). All mixtures buffered at pH 8 with M/15 phosphate, 2 drops of chloroform added as bacteriostatic; neutralized, and B.A.L. added to 0·01 M, before test.

Figures represent reciprocals of haemagglutinating titres for rabbit red cells.

B. *Proteolytic Action of Trypsin on Sarcoma Extract in Relation to its Action on the Haemagglutinating Agent, and Inhibition of Both Actions by B.A.L.*

| | Time of incubation 2½ hours. Haemagglutinating titres. | Mg. amino N per 5 c.c., excess over control. |
|---|---|---|
| 1. 20 per cent extract of C57 sarcoma, control | 64 | — |
| 2. The same + trypsin | 0 | 3·43 |
| 3. ,, + ,, + B.A.L. 0·005 M | 64 | 1·82 |
| 4. ,, + ,, + ,, 0·01 M | 64 | 1·61 |

Relevant conditions as in A.

C. *Inhibition by B.A.L. of Tryptic Digestion of Casein.*

| 4 per cent solution of casein, | Time of incubation. 1 hour. | 2½ hours. | |
|---|---|---|---|
| 1. + trypsin | 0·43 | 1·54 | Mg. amino N per 5 c.c., excess over control. |
| 2. + ,, + B.A.L. 0·005 M | 0·57 | 0·84 | |
| 3. + ,, + ,, 0·01 M | 0·43 | 0·56 | |

Casein was "light, soluble" of British Drug Houses, dissolved in M/15 $Na_2HPO_4$ by heating 1 hour on a water-bath.

Relevant conditions as in A.

It will be noted that there was a considerable irreversible drop in titre of a control sample of sarcoma extract incubated without B.A.L. (Table VII A, No. 1), while that of a similar sample incubated with B.A.L. (0·005 M) remained constant (Table VII A, No. 2). A similar drop has been observed during storage at 4° C. of extracts not containing B.A.L., i.e. the titre attainable by addition of B.A.L. to such extracts gradually falls. It seems probable that this effect is due to the action of tissue proteases.

The effect of tryptic digestion on extracts of normal mouse tissues was also tried. It is difficult to obtain such extracts with haemagglutinating titres high enough to make this test satisfactory. In an experiment in which a mouse embryo extract with a haemagglutinating titre for rabbit cells of 1:4 was digested with trypsin, with and without B.A.L., activity appeared to be destroyed in both cases. However, not much reliance can be placed on this result, because haemolysis by trypsin was rapid in the low dilutions, and may have masked haemagglutination.

The results of digestion of tumour extracts suggest that B.A.L. may act as an inhibitor of proteolysis by trypsin and papain. However, Webb and van Heyningen (1947) state that B.A.L. (0·0042 M) does not inhibit digestion of casein by either trypsin or papain, as judged by formol-titration of amino-acid nitrogen, and in certain circumstances activates papain.

Because of this apparent anomaly, the action of B.A.L. on proteolysis was examined, using tumour extracts, and casein, as substrates.

A 20 per cent extract of C57 sarcoma in M/15 phosphate buffer pH8 was incubated (*a*) alone, (*b*) with trypsin, (*c*) with trypsin, and B.A.L. 0·005 M, and (*d*) with trypsin and B.A.L. 0·01 M. The former concentration of B.A.L. was chosen because it has a definite, though sub-maximal, preservative effect on the haemagglutinating agent, and is approximately equal to that used by Webb and van Heyningen, the latter because it has the maximal preservative effect. After 2½ hours the mixtures were tested for haemagglutinating power, and their amino-nitrogen content determined by formol-titration.

In a similar experiment, a 4 per cent solution of casein ("light, soluble," British Drug Houses) in M/15 phosphate buffer pH8 was substituted for the tumour extract. Amino-nitrogen was determined after 1 and 2½ hours' incubation.

The results of these experiments are given in Table VII B and C. They show that, with casein as substrate, after 1 hour's incubation there was no detectable inhibition of proteolysis by B.A.L.; but with both casein and tumour extract as substrates, after 2½ hours' incubation there was definite inhibition: about 50 per cent by B.A.L. 0·005 M, and 60 per cent by 0·01 M. Both concentrations of B.A.L. protected the haemagglutinating agent from the action of trypsin for 2½ hours.

The action of crystalline trypsin, kindly supplied by Dr. S. D. Elliott, on the haemagglutinating power of a tumour extract, with and without B.A.L., was essentially similar to that of commercial trypsin.

The haemagglutinating agent does not become dialysable through cellophane after digestion of a B.A.L.-containing tumour extract with trypsin.

Lecithinase: It has been shown by Stone (1946*a*) that the haemagglutinating action of lipoids, and of vaccinia and ectromelia viruses, which probably owe their haemagglutinating action to lipoid components, is destroyed by *Cl. welchii* toxin, which contains an active lecithinase.

A sample of partially purified *Cl. welchii* toxin was kindly supplied by Miss M. G. Macfarlane. A preliminary test against egg-yolk (the lecitho-vitellin test of Macfarlane, Oakley and Anderson, 1941) showed that this preparation was highly active. The addition of B.A.L. (0·01 M) to the substrate greatly retarded the action of the enzyme, as judged by this test. Accordingly, its action on tissue extracts not containing B.A.L. was tried. To C57 sarcoma extract, buffered at pH 7·6 by the addition of borate buffer, and containing $CaCl_2$ 0·01 M (Stone, 1946*a*), *Cl. welchii* toxin was added to give a final concentration of 1·2 lecithinase units per c.c. (The unit of lecithinase, defined by Macfarlane and Knight (1941) is that amount which, under defined conditions, produces 0·1 mg. acid-soluble P from lecithin in 15 minutes at 37° C.) The mixture was incubated for $2\frac{1}{2}$ hours at 37° C. B.A.L. to 0·01 M was then added, and haemagglutinating power tested. In a similar experiment mouse embryo extract was treated with the toxin.

The results showed that incubation with lecithinase had no detectable effect on the haemagglutinating agents of C57 sarcoma or mouse embryo extracts. Haemolysis by lecithinase present in the mixtures was not rapid enough, in the presence of B.A.L., to interfere with haemagglutination.

*Action of heavy metals and oxidizing agents, and its reversal by B.A.L.*—To samples of fresh, actively haemagglutinating, sarcoma extract, not containing B.A.L., $CuSO_4$ was added, to give final concentrations of M/300 and M/3000. In both mixtures haemagglutinating activity disappeared within a few seconds; there was a partial return of activity in the later, but none in the former, after addition of B.A.L. to 0·01 M. Manganese, cobalt, and ferric salts, had similar but less powerful effects in the same concentrations, which were also partially reversible by B.A.L. In order to determine whether the natural inactivation of the agent is due to the action of heavy metals, samples of a fresh sarcoma extract, not containing B.A.L., were saturated with benzoin-oxime ("Cupron") and 8 hydroxy-quinoline respectively. The former substance reacts specifically with copper, the latter with any heavy metal, forming inactive compounds. The haemagglutinating activity of these mixtures declined on standing at the same rate as that of a control. It thus appeared that the presence of heavy metals was not essential for natural inactivation.

The addition of various oxidizing agents to a fresh sarcoma extract destroyed or reduced its haemagglutinating activity. Hydrogen peroxide (10 per cent of "hydrogen peroxide 100 vols." Boots), sodium hypochlorite (10 per cent of a 1 per cent solution—"Milton"), and iodine (M/300) all destroyed the activity completely, while lower concentrations produced a drop in titre. Activity was restored, to a degree depending on relative concentrations, by the addition of B.A.L.

### DISCUSSION.

A number of substances of widely various type and origin have the power to agglutinate red cells. The list includes serum antibodies (naturally occurring, acquired, or experimentally induced), many viruses (Hirst, 1941; Nagler, 1942; Burnet, 1945; Lush, 1943; Burnet and Stone, 1946; Mills and Dochez, 1944), purified lipoids (Stone, 1946*b*), bacterial extracts (Keogh, North, and Warburton, 1947), the globulin fraction of egg-white (Commission on Acute Respiratory Diseases, 1946), some plant proteins such as ricin and abrin, basic proteins such as protamines and histones, inorganic colloidal acids and bases such as colloidal silicic acid, and many metallic salts (Landsteiner, 1945). No report has appeared, as far as the author is aware, of haemagglutination by extracts of tissues, either normal or neoplastic. Gross (1947, 1948) has described haemolysis of mouse, but not rabbit, guinea-pig, or human red cells, by extracts of mouse mammary carcinoma and lymphatic leukaemia tissue, both spontaneous and grafted, but he does not report the occurrence of haemagglutination. It is perhaps not surprising that haemagglutination by tissue extracts has not been hitherto observed, in view of the lability of the agents. It was a fortunate accident that C57 sarcoma was the first tumour to be examined, extracts of which remain active for several hours. But for this, the phenomenon would probably not have been noticed, for the majority of tumour and normal tissue extracts examined remain haemagglutinating for a much shorter time, and then only for rabbit cells. Their study would not have been possible without experience gained with C57 sarcoma.

The tissue haemagglutinating agents differ from both viruses and lipoids in various ways. They are labile, while the latter are both fairly stable substances ; their red-cell species specificity is different from that of both lipoids and viruses ; they are unaffected by *Cl. welchii* toxin, which destroys the haemagglutinating power of lipoids and of vaccinia and ectromelia viruses ; their action is unaffected by normal serum, which inhibits that of lipoids but not that of viruses. In the manner in which temperature affects haemagglutinating titre they differ from lipoids. A sarcoma extract is absorbed by red cells which it agglutinates, but the cells, when they redisperse, as they do under certain conditions, are fully agglutinable by the same agent ; red cells agglutinated by a virus may also redisperse, but are then inagglutinable by that virus, and often by some others also (Burnet, Beveridge, McEwin and Boake, 1945).

Egg-white is more like the tissue extracts in its haemagglutinating properties than are the other haemagglutinating substances referred to, particularly in respect of red cell species range, and effect of temperature on haemagglutinating titre, but it is not reported to be labile.

The general properties of the haemagglutinating agent of tumours suggest that it is a readily oxidizable substance which is active only in the reduced state. It may be a sulphydryl compound, but there is at present no positive evidence of this. Neither inactivation by heavy metals, nor by oxidizing agents, is conclusive on this point. The former may act by forming compounds with sulphydryl groups, or by catalyzing oxidation ; the latter may act on sulphydryl or other oxidizable groups. More evidence is needed, also, before the nature of the preservative action of B.A.L. and other sulphydryl compounds can be determined. However, the fact that other reducing agents, e.g. ascorbic acid, are ineffective, suggests that preservation is a specific effect of sulphydryl. Natural inactivation does not appear to depend on the presence of heavy metals in an active state, and therefore it is unlikely that its prevention by B.A.L. is due to the latter's power of combining with them.

The protection of the haemagglutinating agent by B.A.L. from destruction by proteolytic enzymes is difficult to understand on the basis of available knowledge. B.A.L. has been stated not to inhibit the action of trypsin or papain (Webb and van Heyningen, 1947), but this work has been repeated here, with a different result. It has been shown that B.A.L. 0·005 M produces 50 per cent inhibition of proteolysis of casein, or tumour extract, incubated with trypsin for 2½ hours. During this period B.A.L. protects the haemagglutinating agent from the action of trypsin, whereas in the absence of B.A.L. it is completely destroyed.

A fuller understanding of these facts must await the result of further work.

Attempts to find qualitative differences between the haemagglutinating agents of normal tissues and those of tumours, or between those of different tumours, have been unsuccessful. The observed differences are either so small as to be of doubtful significance, or can be regarded as quantitative. Though the possibility that different types of agent exist has not been excluded, there is no positive evidence of this at present.

SUMMARY.

Extracts of mouse tumours agglutinate rabbit, mouse, and some other, red cells.

Some normal tissue extracts agglutinate rabbit cells; a few agglutinate mouse cells also. Extracts of mouse embryo, placenta, uterus, testicle, ovary, voluntary muscle, skin, and large intestine, have slight but definite activity, those of mouse brain, heart, lung, liver, spleen, kidney, thyroid, seminal vesicles, and lymph glands, have none.

Rabbit red cells are the most sensitive to agglutination of those tested. Mouse cells are agglutinated to titres four to sixteen times lower than rabbit; sheep, horse, guinea-pig, human, and fowl cells two to four times lower than mouse.

The haemagglutinating agents of tumours and of normal tissues lose their activity rapidly on standing. Addition of sulphydryl compounds, notably 2:3-dimercapto-propanol (B.A.L.) preserves their activity, or regenerates it after loss. Loss of activity is not prevented by addition of other reducing agents, or of enzyme poisons, or by storage in an oxygen-free atmosphere.

Certain physical and chemical properties of the haemagglutinating agent of tumours are described. Reasons are given for regarding it as an easily oxidizable substance, active only in the reduced state. It is destroyed by proteolytic enzymes, but not by lecithinase; B.A.L. protects it from the former.

B.A.L. was found to have definite inhibitory action on proteolysis by trypsin, and on digestion of egg-yolk by lecithinase.

The haemagglutinating agents of tumours and of normal tissues differ from haemagglutinating viruses and lipoids in important respects.

No qualitative differences have been found between the haemagglutinating agents of different tumours, or between those of tumours and normal tissues.

The author gratefully acknowledges his debt to Professor J. R. Marrack for carrying out amino-acid-nitrogen estimations, to Professor R. H. S. Thompson and Dr. C. J. O. R. Morris for valuable suggestions, to Professor A. Haddon and Drs. J. G. Carr, L. Dmochowski, D. Parsons, and C. Hoch-Ligeti for gifts of tumour-bearing animals, and to Professor Thompson for a supply of purified B.A.L.

The expenses of this research were defrayed out of a block grant from the British Empire Cancer Campaign.

REFERENCES.

BURNET, F. M.—(1945) *Aust. J. Sci.*, **8**, 81.

*Idem* AND ANDERSON, S. G.—(1946) *Brit. J. exp. Path.*, **27**, 236.

*Idem*, BEVERIDGE, W., MCEWIN, J., AND BOAKE, W.—(1945) *Aust. J. exp. Biol.*, **23**, 177.

*Idem* AND STONE, J. D.—(1946) *Ibid.*, **24**, 1.

Commission on Acute Respiratory Diseases, Fort Bragg, N.C.—(1946) *Proc. Soc. exp. Biol., N.Y.*, **62**, 118.

GROSS, L.—(1947) *Ibid.*, **65**, 292.—(1948) *Ibid.*, **67**, 341.

HIRST, G. K.—(1941) *Science*, **94**, 22.

KEOGH, E. V., NORTH, E. A., AND WARBURTON, M. F.—(1947) *Nature*, **160**, 63.
LANDSTEINER, K.—(1945) 'The Specificity of Serological Reactions.' Harvard: Univ. Press, pp. 4–7.
LUSH, D.—(1943) *J. comp. Path.*, **53**, 157.
MACFARLANE, M. G., AND KNIGHT, B. C. J. G.—(1941) *Biochem. J.*, **35**, 884.
MACFARLANE, R. G., OAKLEY, C. L., AND ANDERSON, C. G.—(1941) *J. Path Bact.*, **52**, 99.
MILLS, K. C., AND DOCHEZ, A. R.—(1944) *Proc. Soc. exp. Biol. N.Y.*, **57**, 140.
NAGLER, F. P. O.—(1942) *Med. J. of Aust.*, **1**, 281.
SALK, J. E.—(1944) *J. Immunol.*, **49**, 87.
STONE, J. D.—(1946*a*) *Aust. J. exp. Biol.*, **24**, 191.—(1946*b*) *Ibid.*, **24**, 197.
WEBB, E. C., AND VAN HEYNINGEN, R.—(1947) *Biochem. J.*, **41**, 74.

---